# Modulating Surface Properties of the *Linothele fallax* Spider Web by Solvent Treatment

**DOI:** 10.1021/acs.biomac.1c00787

**Published:** 2021-10-13

**Authors:** Aleksandra Kiseleva, Gustav Nestor, Johnny R. Östman, Anastasiia Kriuchkova, Artemii Savin, Pavel Krivoshapkin, Elena Krivoshapkina, Gulaim A. Seisenbaeva, Vadim G. Kessler

**Affiliations:** † Institute of Solution Chemistry of Advanced Materials and Technologies, ITMO University, St. Petersburg 197101, Russia; ‡ Department of Molecular Sciences, Biocenter, SLU, Box 7015, Uppsala 75007, Sweden

## Abstract

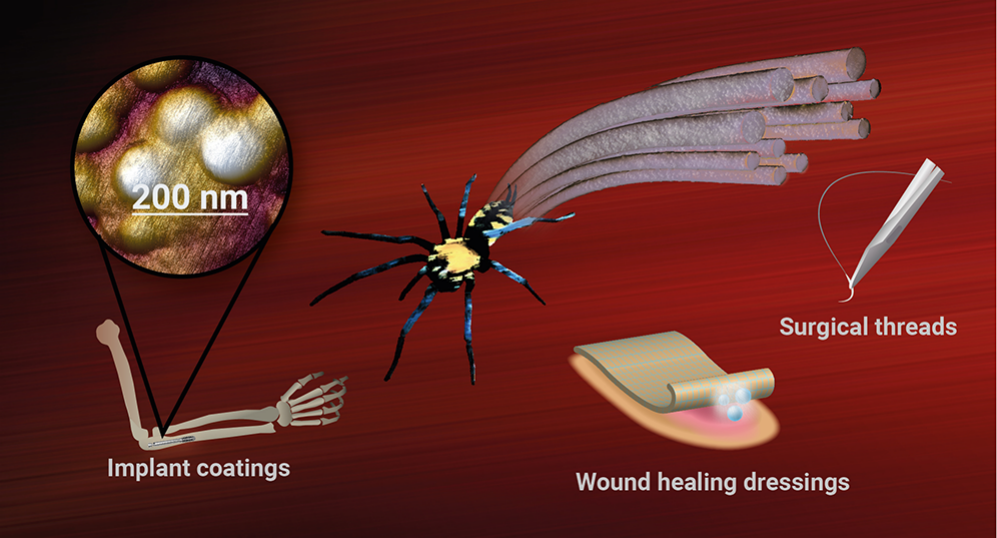

*Linothele fallax* (Mello-Leitão) (*L. fallax*) spider web, a potentially attractive tissue engineering material, was investigated using quantitative peak force measurement atomic force microscopy and scanning electron microscopy with energy dispersive spectroscopy both in its natural state and after treatment with solvents of different protein affinities, namely, water, ethanol, and dimethyl sulfoxide (DMSO). Native *L. fallax* silk threads are densely covered by globular objects, which constitute their inseparable parts. Depending on the solvent, treating *L. fallax* modifies its appearance. In the case of water and ethanol, the changes are minor. In contrast, DMSO practically removes the globules and fuses the threads into dense bands. Moreover, the solvent treatment influences the chemistry of the threads’ surface, changing their adhesive and, therefore, biocompatibility and cell adhesion properties. On the other hand, the solvent-treated web materials’ contact effect on different types of biological matter differs considerably. Protein-rich matter controls humidity better when wrapped in spider silk treated with more hydrophobic solvents. However, carbohydrate plant materials retain more moisture when wrapped in native spider silk. The extracts produced with the solvents were analyzed using nuclear magnetic resonance (NMR) and liquid chromatography–mass spectrometry techniques, revealing unsaturated fatty acids as representative adsorbed species, which may explain the mild antibacterial effect of the spider silk. The extracted metabolites were similar for the different solvents, meaning that the globules were not “dissolved” but “fused into” the threads themselves, being supposedly rolled-in knots of the protein chain.

## Introduction

Natural spider silk is an extremely strong, elastic, and flexible material,^1−5^ which was used for centuries in wound healing.^6−9^ Spider silk shows reasonable biodegradability and biocompatibility,^10−12^ with only negligible inflammatory response. Since this natural protein has combined charged residues and alternating hydrophilic and hydrophobic regions,^13^ it may support the proliferation of different cell types, facilitating cell growth and adhesion.^14−17^ Thus, spider silk is a perspective material for the development of many biomedical applications, such as tissue regenerating, wound healing dressing, implant coatings, artificial tendons, surgical threads, and blood vessel support.^14,18−21^ Spider silk-based biopolymers show a slight antibacterial effect, demonstrated in nature by spiders’ eggs and extra food wrapped in silk fibers. The stored eggs and food are preserved for months or even years unaffected by fungi and other microorganisms.^22,23^ Although several reports attempted to decode the origin of the antimicrobial properties of spider silk, the findings were unconvincing, or the methods used were not accurate enough.^24−26^ The evolutional antimicrobial activity of spider silk is presumably based on surface or structural qualities of the fibers, including physiologically important chemical compounds and micro- and nanotopography features, determining the material interaction with water.^27^ Various antifungal and antimicrobial agents, such as bisphosphonates, peptides, phospholipid hydrates, and potassium nitrate, have been found in silk fibers.^26^ They define the bacteriostatic efficiency of spider silk fibers and demonstrate that the development of drug-free spider silk-based surgical biopolymers may improve antibacterial properties of the biomedical materials by suppression of the microorganisms’ growth or reproduction.^28^ As long as the occurrence of postoperative inflammations composes one-fifth of post-transplant complications, it is crucial to identify the material capable of firmly connecting the places of tissue injuries, providing cell proliferation, preventing formation of biofilms, and, consequently, restricting infections.^29,30^ Several studies suggested that microorganisms are unable to grow on spider silk due to its acidic nature.^31^ Since acidic conditions have been proposed to inhibit the growth of fungi, various pathogens, and silk protein-digesting bacteria, spider silk is expected to be protected from bacterial attack and degradation.^31,32^ On the other hand, various studies revealed that lipids in the spider silk contain several *anteiso*-fatty acids, such as 12-methyltetradecanoic acid and 14-methylhexadecanoic acid, that seem to regulate the water content of silk and protect protein-rich silk from degradation by inhibiting the growth of microorganisms.^33−36^ It is also worth noting that spiders can protect themselves from natural predators by synthesizing specific compounds. For example, *Nephila antipodiana* spiders have been found to produce silk containing an insecticide, 2-pyrrolidinone, which seems to promote self-defense against ant attacks.^37^

The antimicrobial activity observed in the abovementioned studies arises from a combination of soluble/insoluble growth-restraining factors in silk and surface or structural material properties. For example, SCP-1 and SCP-2 small coating peptides have been found on the fiber surface of aciniform and tubuliform spider silks. They perform functions of antimicrobial agents that help to prolong the longevity of the fibers.^38^ Additionally, other components of spider silk, including phosphorylated glycoprotein, inorganic salts, pigments, sulfur-containing amino acids, ionic forms of amines, and lipids, are physiologically important and act as a protection layer in the fibers and enhance the adhesiveness of silk fibers.^39,40^ Upon contact with a solvent, spider silk releases antibacterial substances into the medium, inducing antimicrobial and inhibitory effects on a diverse group of microorganisms.

The properties and composition of silk vary noticeably between and within spider species.^41^ Therefore, some discrepancies in response to silk from different spider species should be expected. Additionally, different types of silk have various amino acid compositions.^42^ Because of the hygroscopic nature of these amino acids, they prevent silk from drying out by absorbing moisture from the air and saving it within the fiber.^43^ This unique peculiarity is observed in the structure of silk produced by the *Uloborus walckenaerius* spider, featuring spindle-knots made of random and aligned nanofibrils, forming superhydrophobic and superhydrophilic patterns. The latter play a key role in water collecting from a humid atmosphere on a micro- and macroscale.^44^

Wettability (hydrophilicity/hydrophobicity) is a material surface property, which affects bacterial adhesion, inducing special qualities, such as self-sterilization to a surface.^45^ Both negatively charged superhydrophobic and superhydrophilic surfaces have been reported to be self-cleaning and, therefore, unsusceptible to bacterial adhesion.^46,47^ Of particular note, surface wettability is determined by micro- and nanoscale surface topography. Thus, surface roughness influences superhydrophobicity that limits bacterial adhesion to the surface.^45^ In parallel, a lot of other natural biomaterials exhibit similar remarkable surface characteristics. For instance, brush-turkey eggshells are covered with calcium phosphate nanospheres,^48^ which create a rough superhydrophobic topography that results in low bacterial adhesion, contributing to the antimicrobial defense of the eggs.^49^

Investigation of silk features as a nanocomposite material contributed to understanding complex architectural designs of nanocrystals (∼2–4 nm) and self-assembly of nanofibrils (∼20–80 nm).^50,51^ Nanofibrillar organization is assumed to play an essential role in the native spider silk “core” structure. However, insights into its hierarchical “skin” architecture and the full complexity of its constituents remain elusive. Investigation of the hierarchical organization of the spider silk fiber into distinct structural elements based on various morphological features of this material demonstrates an inner core discriminated from outer skin layers covered with nanosized fibrils.^52,53^ The origin and functions of the nanoscale patterns found on the surface of native spider silk have remained undetermined. To date, five different silk layers have been distinguished, which can essentially influence the mechanical performance and distinctive properties of spider silk. However, only two of them are core layers and contain known silk proteins.^54^

The initial premise of this work assumed that the biomedical properties of spider silk could be explained by the presence of some specific compounds in the skin layers of the fibers. Conversely, we observed that upon contact of native spider silk with different solvents, fiber morphology changed considerably, and the strength of surface interactions increased. To understand this phenomenon, a comprehensive approach was required. Previous attempts to understand structure–property relations in spider silk utilized nuclear magnetic resonance (NMR),^55,56^ mass spectrometry (MS)^57^ and X-ray diffraction,^58,59^ optical,^54,60^ electron,^53,61−63^ and atomic force microscopy (AFM).^52,64,65^ Recently, AFM has been beneficial for understanding the structure–property relationships in spider silks since AFM allows us to investigate the surface structure, interactions, and local mechanical properties.^66,67^

While research on the silk complex structure is relatively widespread, the components which play a key role in enhancing the surface interactions, including adhesion to bacteria or cells, remain elusive. Therefore, the purpose of this work was to provide straightforward analytical understanding of the chemical nature of the healing and antimicrobial effects of native spider silk and to link morphologically defined structural elements of spider silk’s surface to its biochemical composition and physicochemical properties. In the present study, we demonstrated that nanopatterns covering the native spider silk surface are central for surface interactions. Additionally, we identified the mechanisms of conformational changes, depending on the interaction with solvents, which brought insights on the molecular level. Specifically, the present study aims were to shed light on the mystery of the nature of surface globules of spider silk threads and to get insights into how their principal functional characteristics emerge. Explanation of the relationship between the supramolecular organization of spider silk and the origin of its outstanding biological properties is expected to open avenues in the production of high-performance artificially spun fibers.

## Experimental Section

### Materials

Ethanol (96.0–97.2%, Sigma-Aldrich), dimethyl sulfoxide (DMSO) (VWR chemicals, Molecular Biology Grade), and Milli-Q grade deionized water (18.2 MΩ·cm^–1^ resistivity) were used in all experiments. Materials used for antibacterial tests included Luria–Bertani (LB) agar (microbiologically tested, Sigma-Aldrich).

*Linothele fallax* (Mello-Leitão) (*L. fallax*) spider (see Figure S1) web harvesting was done from individuals fed for 2–3 weeks prior to silk collection. *L. fallax* is a species of spiders from the family *Dipluridae*, known as curtain-web spiders in the suborder *Mygalomorphae.* They have very long spinnerets and build silk-lined burrows instead of circular spider web produced by many other orders of spiders.^68−70^ An important feature of spider species in the suborder *Mygalomorphae* is that this order possesses a comparatively undifferentiated spinning apparatus consisting of uniform spigots that lead to homogeneous, acinous-shaped silk glands and generate one single type of silk thread woven into dense mats, making it an attractive model for studies.^71−73^

Treatment with solvents was carried out on ca. 50 mg of spider silk with 2 mL of solvent in glass vials by shaking overnight. The web was then separated using tweezers and dried for 24 h in air on a Petri dish and then again for 24 h on carbon tape for subsequent scanning electron microscopy (SEM) and atomic force microscopy (AFM) studies.

### Methods

#### Fourier Transform Infrared Spectroscopy (FTIR)

FTIR analysis was used to investigate the silk’s secondary structure. Spectra were acquired with a Nicolet iS10 FTIR spectrometer (Thermo Fisher Scientific). The spectra were measured in the range from 4000 to 400 cm^–1^ in the absorbance mode. Dry samples were treated with crystalline KBr (1 mg of sample:199 mg of KBr) and then pressed into a disk. The data were obtained at a resolution of 0.5 cm^–1^ with 40 cumulated scans and a signal-to-noise ratio of 40,000:1. The fitting of the amide I peak was performed using Gaussian functions. The criteria employed for the determination of the initial conditions of the fitting were based in the second derivative of the experimental spectrum. The minima of the second derivative were used to determine the number and position of the Gaussian functions used for the fitting. The initial full width at half maximum was from the beginning fixed to 8 cm^–1^ for every Gaussian used to fit the spectrum. Finally, the fitting was performed after applying a linear baseline. The fitting process was in all cases carried out with Omnic 9 software; please see the example of the deconvolution process in Figure S2. The areas of the Gaussians were used to estimate the content of the different secondary structures according to the band assignments, which are presented in Table 1.

**Table 1:** Vibrational Band Assignment in the Amide I Region

wavenumber^a^	assignment	references
1621–1627	intermolecular β-sheet	(74−77)
1628–1637	intramolecular β-sheet	(74, 76, 78−80)
1638–1655	random coil	(78, 79)

a Unit in wavenumbers (cm^–1^).

#### Atomic Force Microscopy

AFM studies were carried out on a Bruker Advance FastScan Bio with a ScanAsyst microscope, equipped with QPFM hardware (cantilever calibration readout unit) and software. The cantilevers applied were precalibrated with a Bruker RTESPA-150-30 (Antimony(n)-doped Silicon). Calculations for analyzing peak force curves were made using the Bruker AFM Analysis platform and the SPIP program (Ref: Scanning Probe Image Processor software by ImageMet A/S Denmark, https://www.imagemet.com/products/spip/).

#### Scanning Electron Microscopy with Energy Dispersive Spectroscopy

SEM–energy dispersive spectroscopy (EDS) investigation was done using Hitachi TM-1000-μ-DeX and Flex-SEM-1000 II microscopes. EDS analysis was carried out with an Oxford Instruments AZtec Live system. The samples were produced by attaching a portion of a web on a piece of thin carbon tape (Hitachi High-Tech Sweden) attached in turn to a steel disc. Identical samples were used in both AFM and SEM, but in the latter case, they were also sputtered with gold in 1 min. This explains the presence of traces of Fe in the analysis and also of Au (removed from quantification).

#### Nuclear Magnetic Resonance

NMR spectra were acquired on Bruker Advance III 600 MHz spectrometers with a 5 mm inverse detection cryoprobe or a 5 mm broadband observe detection SmartProbe, both equipped with a *z* gradient. Extracts for NMR were prepared by treating spider silk (50 mg) with deuterium oxide (D_2_O), ethanol-*d*_6_, or DMSO-*d*_6_ in glass vials overnight. Spectra from the D_2_O extract were acquired with a 1D NOESY experiment with presaturation (Bruker pulse sequence *noesygppr1d*) to suppress the residual water signal. Spectra were recorded at 25 °C and were processed with TopSpin 3.6.1. ^1^H chemical shifts were referenced to solvent signals (HDO 4.77 ppm; ethanol-*d*_6_ 1.11 and 3.56 ppm; and DMSO-*d*_6_ 2.50 ppm). Signals were assigned with the help of TOCSY and HSQC experiments.

#### Liquid Chromatography–Mass Spectrometry

LC–MS analysis was carried out on water and ethanol extracts. The chromatographic separation was performed on an Agilent 1290 Infinity II (Agilent Technologies, Santa Clara, CA) LC using a Waters Xbridge BEH amide column (3.5 μm, 4.6 × 100 mm). The aqueous phase consisted of 95:5 (vol/vol) water/acetonitrile with 20 mmol/L ammonium acetate and ammonium hydroxide. The separation was carried out using gradient elution. The following gradient program was used (% organic phase): 0 min 85%, 3 min 30%, 12 min 2%, 15 min 2%, 16 min 85%, and 23 min 85%. A total of 10 μL of the extract was injected onto the column thermostatted at 30 °C, and the mobile-phase flow rate was 400 μL/min. The MS analysis was performed on a Bruker maXis Impact (Bruker Daltonics, Bremen, Germany) high-resolution time-of-flight mass spectrometer with an electrospray ion source. The capillary voltage was set at 4 kV with a plate offset of 500 V. Desolvation was done using 200 °C nitrogen gas at 8 L/min and a nebulizer pressure of 2 bars. The sampling rate was 4 GHz, and profile mass spectra were collected at a rate of 1 Hz. Mass-to-charge ratios (*m*/*z*) corresponding to unsaturated, monounsaturated, and di-unsaturated carboxylic acids and saturated and monounsaturated dicarboxylic acids with chain lengths from 3 to 36 carbons were screened. The *m*/*z* was considered to be present in the sample if the extracted ion chromatogram showed a local signal-to-noise ratio > 3.

#### Cell Adhesion Experiments

Nonmalignant hFB-hTERT6 human skin fibroblasts were obtained via lentiviral transduction of the full-length TERT gene under a cytomegalovirus promoter (donated by Dr. E. Dashinimaev, Engelhardt Institute of Molecular Biology, Moscow). Spider silk samples were exposed to UV light (250 nm) for 30 min before cell seeding for sterilization. Glasses covered with silk were transported in 6 well-plate Eppendorf 200,000 cells (fibroblasts) per well, with samples on the cover glass added. Cells were cultured in Dulbecco’s modified Eagle’s medium supplemented with 10% fetal bovine serum, 50 U mL^–1^ penicillin, and 50 μg mL^–1^ streptomycin at 37 °C and 5% CO_2_ in a humidified atmosphere for 24 h. It is time for cells to adhere to the surface and not start actively proliferating. After that, the cells were washed with 1× phosphate-buffered saline (PBS) (Gibco, 1×, pH = 7.4) and stained with acridine orange (0.005 mg/mL) for 10 min. After washing, the samples were fixed in 4 wt % paraformaldehyde solution in PBS for 10 min and mounted in the glycerol onto the glass slides. The microscopy images were taken on an inverted Microscope Leica DMi8 using 10× and 20× objectives. Acridine orange was used as a stain for better cell visualizing. Fluorescent images were obtained in a rhodamine filter (silk has its fluorescence in every channel partly because of unspecific staining with acridine orange and partly because of its natural properties; RNA molecules in fibroblasts were visualized in a rhodamine filter). For each condition, four distinct samples were analyzed from three independent experiments (plus control samples, see Figure S3).

#### Biological Activity Tests

Biological activity tests were performed in a Petri dish with LB agar. Bacterial culture *Staphylococcus aureus* (*S. aureus*) with a sample of natural spider silk was incubated for 24 h at 37 °C in liquid nutrient medium. The test substance was applied with sterile discs (*d* = 6 mm) with further incubation for 18 h at 37 °C. Later, the inhibition zone of bacterial growth was measured.

#### Degree of the Food Product Freshness

The degree of the food product freshness was determined by bacterioscopy. We used initial sausage samples and samples wrapped in spider webs in square-shape pieces with a 1 cm side. After removing the spider silk, we made uncontaminated slices of the samples, which were applied three times to the slide to obtain an imprint on the glass surface. The number of bacteria was established on Gram-stained^81^ samples using a microscope.

## Results and Discussion

### Morphological Characteristics

**Figure 1:**
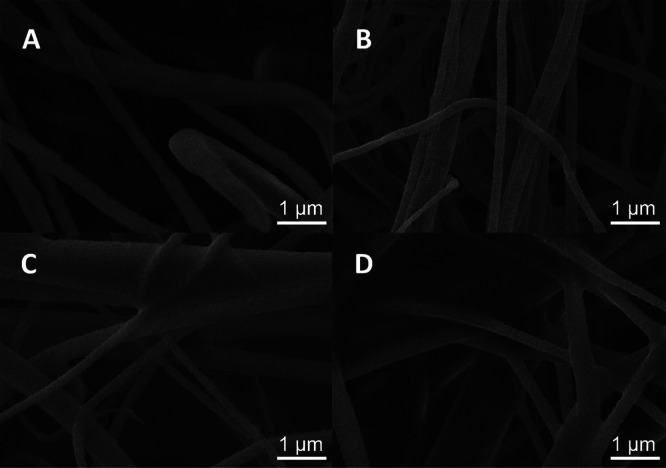
SEM images of the *L. fallax* spider silk: (A) native spider silk and (B) spider silk after treatment with water, (C) ethanol, and (D) DMSO.

The thickness of the *L. fallax* web silk threads varies from 0.2 to just below 2 μm according to SEM imaging (Figure 1) and is not visibly affected by treatment with solvents. Its chemical composition, determined by EDS, consists of carbon, nitrogen, and oxygen and shows glycine, alanine, and serine as the major building units of the spider silk protein threads.^45^

**Figure 2:**
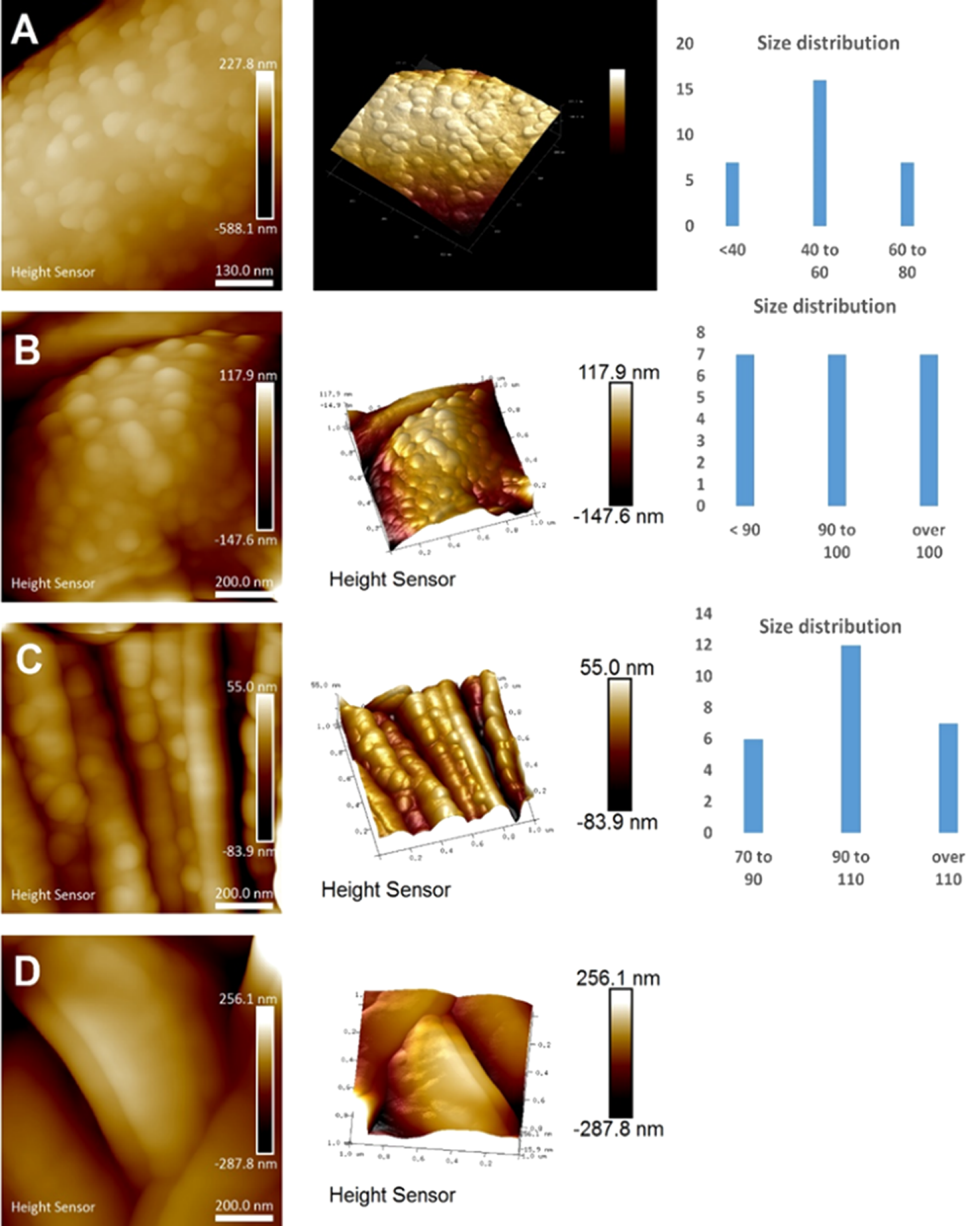
AFM images and size distribution for surface globules in (A) native spider silk and (B) spider silk after treatment with ethanol, (C) water, and (D) DMSO.

A common feature of the threads is their dense coverage by globules, practically forming uniform hexagonal packing on their surface. The size of the globules on the surface of native silk is ca. 40–80 nm, with an average of 56.5 nm (Figure 2A). Submersion in water or ethanol does not change the general appearance of the threads. The size of the globules is, however, greatly affected by the treatment. After immersion in ethanol, the size of the globules increases to ca. 80–110 nm, with an average of 85.5 nm (Figure 2B). After longer exposure to water, the average globule size was 70–120 nm, with an average of about 105 nm (Figure 2C). Conversely, the spider silk threads basically completely lose the globular features after treatment with DMSO (Figure 2D).

The changes in surface appearance are closely connected to the surface forces emerging when interacting with a cantilever. The forces were measured on top of the globules and averaged statistically. For force mapping, please see Figure S4. Native silk displays minimal interactions, with a snap-in force of 27 ± 6 nN. The interaction increases after treatment with ethanol (50 ± 9 nN), becomes even stronger after treatment with water (71 ± 12 nN), and is strongest after treatment with DMSO (121 ± 11 nN).

### Chemical Composition of the Surface

The infrared (IR) spectrum of natural untreated silk showed that vibrational C=O stretching affected the amide I (1600–1700 cm^–1^), and N–H bending belonged to the amide II (1600–1500 cm^–1^) regions of proteins. The bands in the range of 1640–1650 cm^–1^ appear because of stretching vibrations within the C–O and N–H groups that are defined by the conformational structure of the protein backbone.^78,79^ The amide I mode connected with the random coil conformations provides bands in the range of 1640–1650 cm^–1^, and the peak between 1620 and 1640 cm^–1^ represents the β-sheet conformation. After treatment with the DMSO solvent, a new strong band at 1626 cm^–1^ appeared in the spectra instead of the main amide I peak 1652 cm^–1^ of the native spider silk. Because the absorbance at 1626 cm^–1^ is related to the β-sheet conformation of spider silk, the development of the amide I band shows a drastic increase with the changing of solvents from water and ethanol to DMSO. This occurs because solvents are disrupting intramolecular hydrogen bonds between peptide groups, resulting in partial unfolding of globular structures. Despite the lack of hydrogen bonding in organic solvents, they also have electrostatic polar interactions, which contribute to the detected amide I intensity. The intensity variations can be caused by dielectric interactions with the solvent, not by hydrogen bonding alone. The methylene groups (CH_2_) in fatty acids stretch asymmetrically and symmetrically in the range of 2920–2850 cm^–1^ (precisely at 2926 and 2853 cm^–1^, respectively, for asymmetrical and symmetrical stretching) after treatment with ethanol.

The C–N stretching vibration depending on the N–H in-plane bending vibration reveals amide III. The strong O–H absorption of hydroxyl residues of serine and threonine amino acids significantly overlaps the N–H stretching absorption of amide groups with the spectrum at 3300 cm^–1^. The 1394 and 1065 cm^–1^ peaks are assigned with C–OH stretching vibration and C–H and O–H bond vibrations because of the prevalence of hydroxyl amino acid side chains.

**Figure 3:**
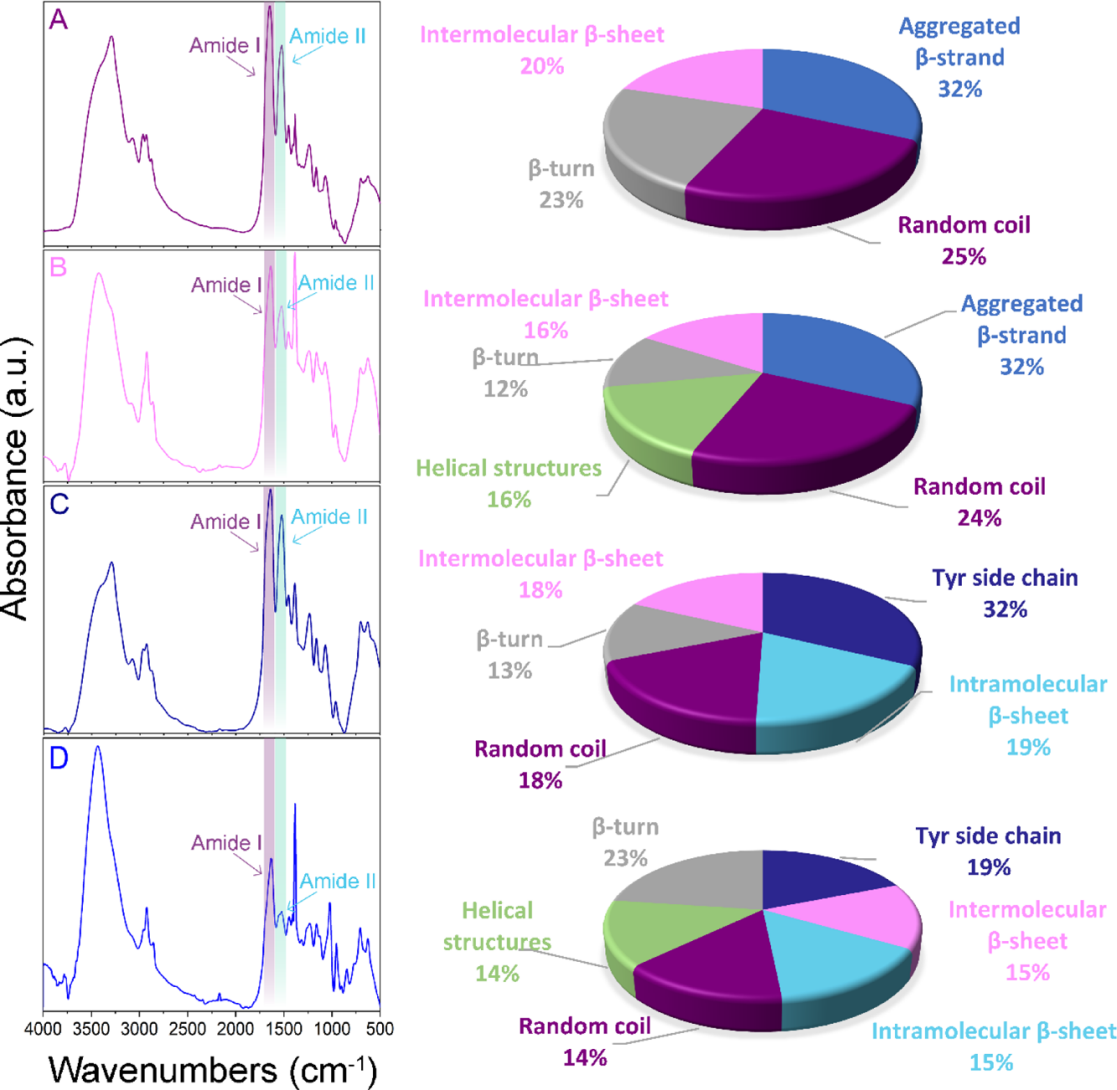
FTIR spectra of *L. fallax* spider silk and percentage of secondary structures in spider silk proteins: (A) native silk, (B) after treatment with ethanol, (C) after treatment with water, and (D) after treatment with DMSO.

The transmission band at 2900–3000 cm^–1^ appears to correlate to alkyl fragments (−CH_3_ and −CH_2_), whereas the peaks of medium intensity at 3330–3070 cm^–1^ in the IR spectrum of the sample appeared to refer to the bending vibration of the imide group (−CONHR). A comparison between native silk and the one treated with ethanol is provided in Figure 3, showing the absence of changes in both the position of bands and their relative intensity. EDS analysis of the samples (see Figure S5a–d) confirms the constant total elemental composition of the web through all kinds of treatment, featuring C, N, and O in the ratio, corresponding to the average for the constituting amino acids (mostly, glycine, alanine, and serine). Elemental maps for these three elements show the same features following the location of spider web threads. It is important to mention that no significant mass fraction of heavy metals was found on the surface of silk thread, eliminating their possible effect on the antibacterial properties of the web.

### Composition of Extracts by Different Solvents

**Figure 4:**
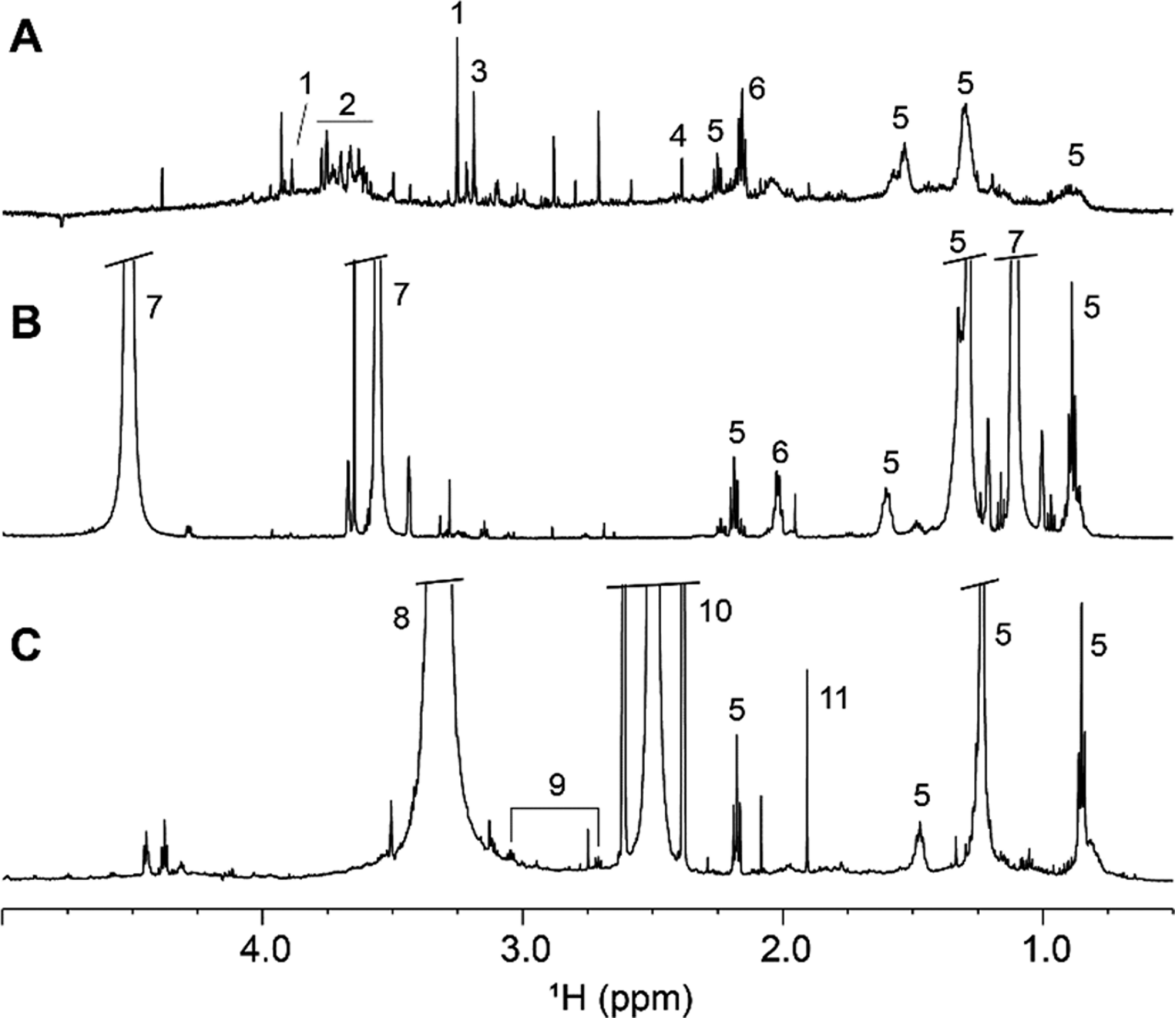
Selected region of ^1^H NMR spectra from (A) water, (B) ethanol, and (C) DMSO extracts of *L. fallax* spider silk. Assigned signals are highlighted with numbers that refer to the different compounds: (1) betaine, (2) glycerol, (3) choline, (4) succinate, (5) fatty acids, (6) unassigned impurity, (7) residual ethanol signals, (8) water, (9) taurine, (10) residual DMSO signal, and (11) acetate. The intensity scale of spectrum A is considerably increased compared to the other two spectra.

NMR spectroscopy was used to trace the species removed from the web by applied solvents. The water extract contained miniscule amounts of low-molecular weight compounds, where choline, glycerol, succinate, and betaine could be detected (Figures 4 and S6). These are most probably residual components of the silk,^88^ partly removed from it by base pretreatment prior to extraction. The ethanol extract, on the other hand, contained mainly saturated fatty acids. The presence of fatty acids in spider silk and spider web droplets has previously been demonstrated.^36,89^ Finally, the DMSO extract showed the presence of saturated fatty acids and small amounts of taurine and acetate.

MS was used to further investigate the spider silk extracts. NMR analysis indicated the presence of fatty acids in the extracts. A range of mass-to-charge ratios corresponding to saturated and unsaturated carboxylic acids with carbon chain lengths between 3 and 36 comprising saturated, monounsaturated, and dicarboxylic acids were thus investigated. Mass-to-charge ratios corresponding to saturated carboxylic acids of all chain lengths from 3 to 26 carbons could be detected. For monounsaturated carboxylic acids, all chain lengths from 4 to 26 carbons could be detected, and for di-unsaturated carboxylic acids, mass-to-charge ratios corresponding to all chain lengths from 5 to 24 carbons could be seen. Signals corresponding to dicarboxylic acids were also detected, with signals corresponding to saturated dicarboxylic acids with chain lengths from 3 to 34 and monounsaturated dicarboxylic acids with 4–24 carbon atom chains being detected. No significant differences in fatty acid species distribution between the water and ethanol extracts could be detected. For details, please see Table S1.

### Biological Characteristics of Materials

Contact antibacterial properties were tested by following the cultivation of *S. aureus* Gram-positive bacteria on Petri dishes. No pronounced clearing zone could be observed around the pieces of the spider web. However, the pressed pieces themselves were not colonized by *S. aureus* Gram-positive bacteria, while single threads were overgrown by them (Figure S7). As almost no bacterial colonies formed on LB medium in the presence of the pieces of the spider web, slight antibacterial activity of the silk threads could be expected.

**Figure 5:**
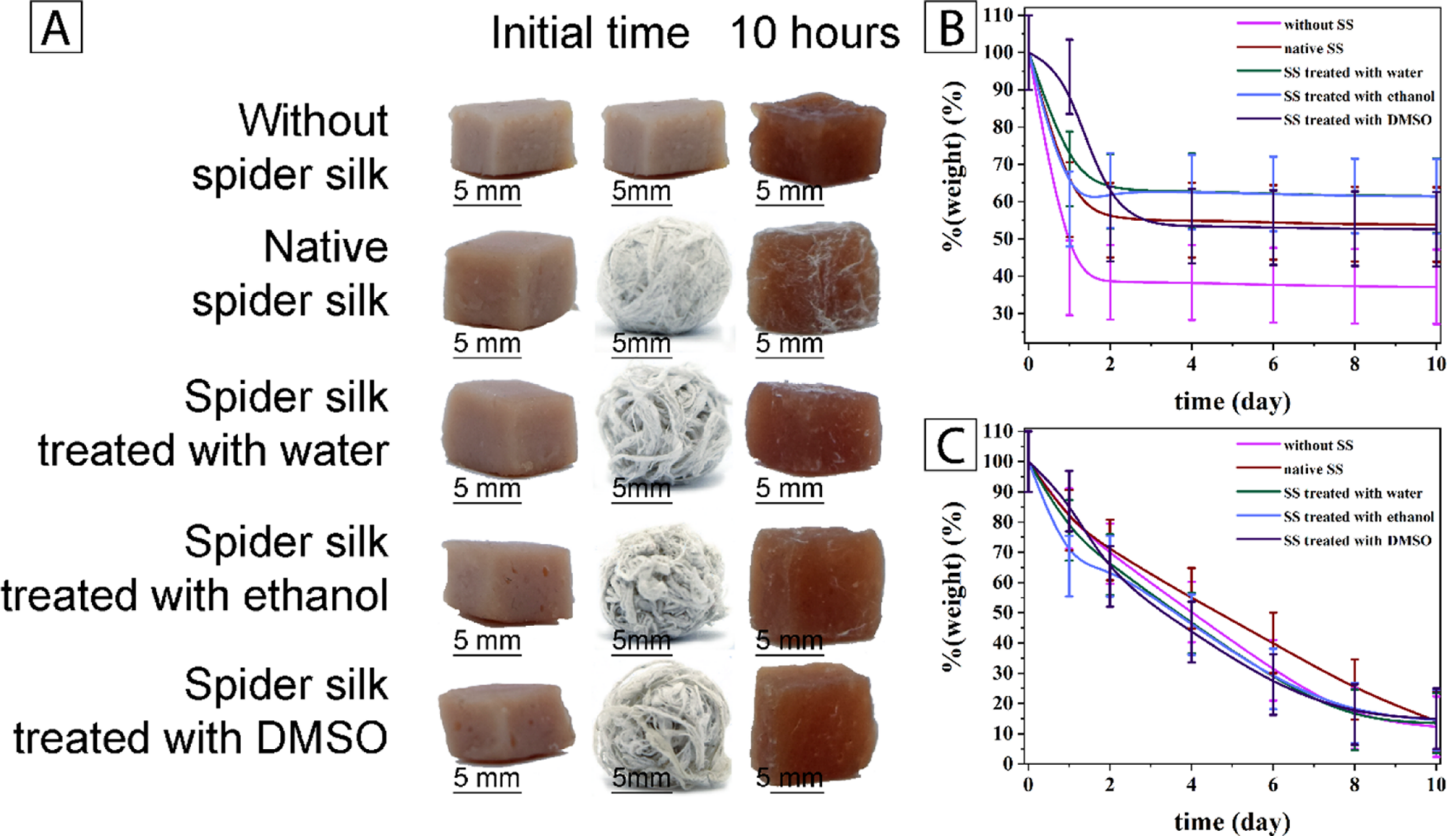
(A) Images of drying of model animal tissue samples, loss of humidity in (B) sausage and (C) fresh raspberry piece weight loss without spider silk and wrapped into native spider silk and into spider web treated with different solvents.

In the search to distinguish the effects of altered surface chemistry on biological systems, we aimed to follow the influence of spider web wrapping on the storage of fresh materials representing animal and plant tissues. The target samples were selected as a 5 × 5 × 5 mm freshly cut piece of Varionaya cooked sausage supplied by the Vostryakovo-2 company, which includes the following ingredients: pork, beef, pork fat, water, egg product (mélange), skimmed milk powder, salt, and extracts of natural spices (black pepper, ginger, and nutmeg). The samples of fresh ripe raspberry, grown in Morocco and produced by Driscoll’s Du Maroc company, were approximately 12 mm in diameter. The surface of each sample was entirely covered by several layers of the spider silk treated with different solvents and native spider silk, and the comparison samples were maintained uncovered. The appearance of animal samples was documented after 10 h (Figure 5A). Sample weight of the model animal and plant tissues was measured every 24 h for 10 days. The tests included a set with five replicates for each product. A distinct difference was observed between the model protein and the plant tissue. Figure 5B,C demonstrates reducing product weight of animal and plant origin with and without wrapping within 10 days. It is important to mention that silk coverage retained its initial appearance and prevented formation of the mold in the case of strawberry, as shown in Figure S8. The view of the internal state of samples was not indicative because of too long storage with the following excessive leakage of water.

For the pieces of sausage, the loss of humidity on drying was distinctly dependent on the nature of treatment for the spider web. The unwrapped material lost over 60% of its weight within a day of storage. Being wrapped in the untreated web led to a loss of about 50%, while using DMSO-treated silk only retained a bit over 10%. However, most notable was that the water- and ethanol-treated webs managed to preserve almost half the moisture content for the entire duration of the experiment (10 days). On the contrary, for the plant material, moisture loss was analogous within the experimental error for both wrapped and unwrapped samples, independent of the solvent treatment.

The degree of product freshness preservation was evaluated using bacteriostatic analysis (Figure S9). Corresponding to the humidity retention, the number of bacteria detected on the pieces of sausage during storage was influenced by the nature of treatment for the spider web. The highest content of bacteria was observed in unwrapped samples. Being wrapped in untreated and water-treated spider silk, the sausage pieces remained the freshest, contaminated almost half as much as unwrapped pieces on the fourth day. Wrapping of samples with ethanol- and DMSO-treated spider webs also reduced the contamination, keeping food fresh, in contrast to unwrapped products.

**Figure 6:**
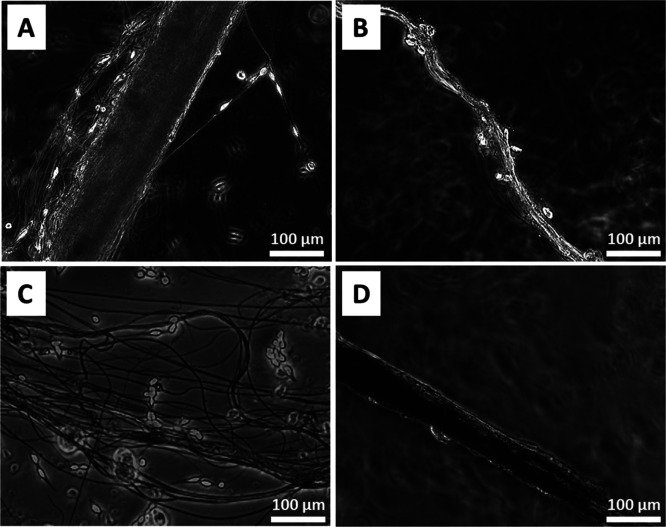
Images of fibroblast cell adhesion tests on scaffolds made from (A) native spider silk and (B) spider silk after treatment with ethanol, (C) water, and (D) DMSO.

Finally, biocompatibility and cell adhesion tests were carried out for nonmalignant hFB-hTERT6 human skin fibroblast cell cultivation on the untreated and solvent-treated spider silk scaffolds. Cell attachment was observed for samples of native silk and silk after treatment with ethanol, after treatment with water, and after treatment with DMSO, in contrast to control tests (see Figures 6 and S3).

### Discussion

*L. fallax* was chosen as the producer of the model material in this work because its silk was recently demonstrated to be attractive as a tissue engineering material with beneficial biointernalization characteristics.^82^ It is a Bolivian curtain-web spider with an enlarged abdomen that makes up half of the spider’s body. Thus, the amount of silk produced is substantially larger than that of typical orb-web spiders.

**Figure 7:**
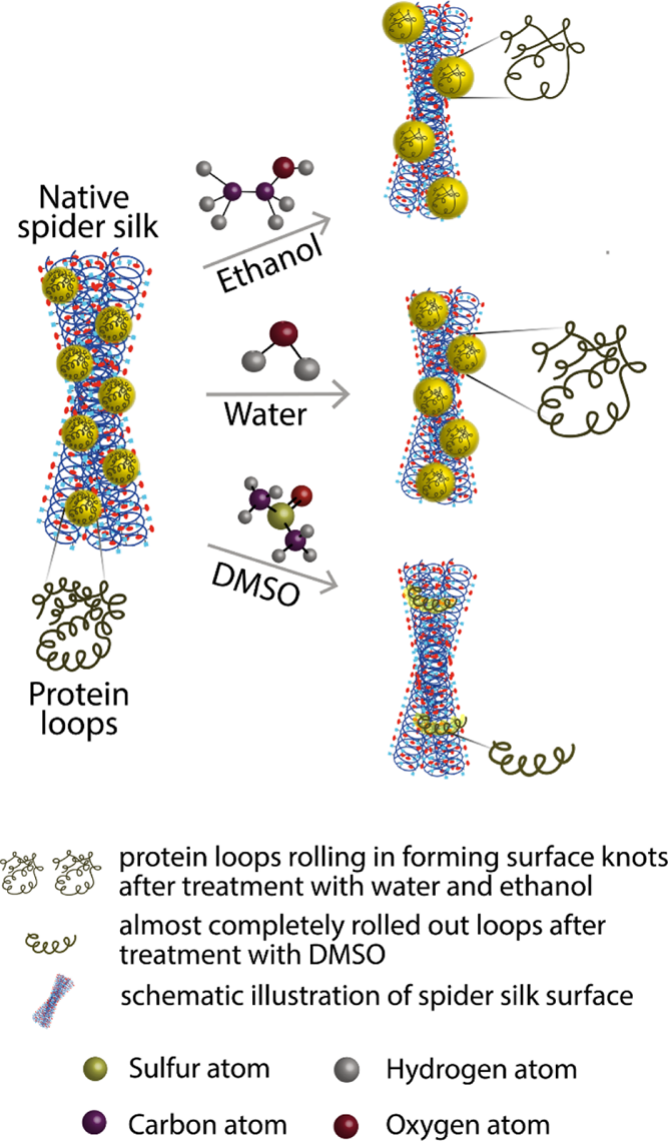
Illustration of protein loops rolling in, forming surface knots.

Apparently, treatment with a solvent does not lead to any noticeable change in the chemical structure, as shown by FTIR investigation, but the availability of different fragments and residues becomes influenced. It can be concluded that the solvating ability of the solvent^74^ and general protein solubility in the said solvent led to an increase in the size of the globules and their eventual disappearance in the case of DMSO.^83,84^Figure 7 illustrates a plausible explanation for the nature of the observed globule morphological structure represented in rolled-in loops of a protein chain, which change in sizes and roll out with varying degrees due to the type of the solvent. This supposition is well in line with the hypothesis proposed recently by Wang and Schniepp^67^ that the single-protein chains in the spider silk are forming knots which contribute to the increased mechanical strength of the threads. Treatment with different solvents influences the ability of threads to interact with other materials. More solvating media render them stickier, which may have importance in surgical thread and tissue engineering applications. Dragline silk transformation on different treatments is a topic of special attention in modulating its properties for biomedical applications.^58^

It can also be concluded that the globules in the case of *L. fallax* spider silk are not glued particles or external protein crystals on the surface of a thread but loops of the threads themselves. The globules were thus not dissolving and releasing some content but supposedly rolling out, forming more open loops with increased capacity for surface interactions, guided apparently by hydrogen bonding. Based on FTIR and NMR analysis, the dissolved content mainly consisted of different *anteiso*-fatty acids and low-molecular weight compounds, including choline, glycerin, succinate, and betaine, supposedly adsorbed on the whole surface of protein threads (for details, please see Table S1). The amount of the material that is dissolved is rather minor, and it mainly contains saturated and unsaturated fatty acids, contributing potentially to the minor antibacterial effect of spider silk. In the view that the material used in this work was pretreated first with a base and then with an acid, the amount of this natural antiseptic was apparently decreased, leading to the absence of a clearing zone in experiments with *S. aureus* bacteria.

The differences between cell attachments were minor and within the margin of error. The reason for the small influence of treatment on cell attachment is the absence of specific amino acid adhesion motifs native for the fibroblast’s environment such as arginyl–glycyl–aspartic acid (fibronectin)^85^ and GFOGER (collagen I)^86^ in most naturally derived silks. The adhesion which we could observe in this experiment depends only on quite weak cell–silk interactions: silk surface charge, wettability, and fibrous topology.^87^ This phenomenon can also be explained by the amphiphilic properties of the globules. As we assume, the globules are similar in nature to the top layer of silk threads, which, according to the data obtained, can bear adsorbed lipids. Untight distribution of globules on the native spider silk suggests that the knot coat will not significantly contribute to the mechanical performance of the fibers. However, rolling out of knots may increase the hydrophobicity of the silk surface, thereby impairing the adhesion of cells, leading to their slithering from the surface of the fibers.

This property also can partially explain the effect of retaining humidity. No influence on the plant tissue rich in and protected by carbohydrate polymers could be observed. On the contrary, preservation of freshness of the protein-rich animal model tissue was more efficient when samples were wrapped with spider silk. The most significant effect on product preservation was achieved by wrapping protein tissues with spider silk fibers after the ethanol treatment. Almost the same strong effect was observed when silk threads were treated with water due to still enlarged sizes of protein knots. Wrapping with the sticky DMSO-treated material protected samples slightly worse. The most inconsiderable influence on protein product saving was observed after wrapping with native spider silk. These effects may be important for tissue scaffolds in wound healing, where humidity balance is crucial, and for the development biodegradable natural food packaging Furthermore, we suppose that spider silk can be used as a coating to enhance the freshness of perishable protein products by deceleration of their respiration, extending protein firmness and preventing dehydration. Wrapping food with silk mats is a potential alternative for preservation, exploiting naturally generated material, permitting water-based processing of comestibles. According to slight antibacterial properties of spider silk, this material can be applicable in packaging without applying other antimicrobial agents. The property of spider silk polymorphism may be applied to modify the coating’s characteristics, altering the effect of silk fibroin on water evaporation and food degradation. Other advantages of spider silk-based packaging are the edibility of silk and possibility to improve coatings with stable biomacromolecules. This expands the usability of functional coatings, which may be used even to provide therapeutic functions to consumable items without resorting to complicated chemistries and enabling preservation.

## Conclusions

In the present work, the surface properties of spider silk were quantitatively characterized in its natural state and after treatment with solvents of different protein affinities—water, ethanol, and DMSO. Native silk threads are densely covered by globular objects, which constitute their inseparable parts. Treatment with the solvents modifies their appearance and adhesive properties, dependent on the solvent. In the case of water and ethanol, changes were minor. In contrast, DMSO practically removed the globules and fused the threads into dense bands.

Most importantly, treatment with the solvents altered the surface chemistry of the threads. The extracted metabolites were similar for the different solvents according to NMR and LC–MS data, implying that the globules were “fused into” the threads themselves, being supposedly rolled-in knots of a protein chain. Treatment with different solvents influences the ability of threads to interact with other materials. These results make an important contribution to the fundamental study of natural scleroprotein-based biopolymers and the development of promising biocompatible materials for medical applications, especially for wound healing. Complete comprehensive wound management usually includes the following aspects: protection of the wound against bacterial activity, control of humidity balance and inflammation, and supporting the growth of the new epithelium. Nevertheless, modern methods of therapy cannot simultaneously provide for all these needs. Since solvating media rendered spider silk fibers stickier and enlarged the size of the globules that regulate humidity balance and cell adhesion, the obtained data may cause a great interest in the development of wound healing dresses and in creation of surgical threads and for tissue engineering. Spider silk-based surgical materials not only meet mechanical and functional application requirements and induce an immune response but also overcome the formation of bacterial biofilms because of a slight antibacterial effect, reducing the risk of infections.

## Supporting Information Available

*L. fallax* spider image, image of the process of native spider silk deconvolution in Omnic 9 software, adhesion force mapping of the samples, ^1^H NMR spectra from *L. fallax* spider silk, image of *S. aureus* Gram-positive bacterial growth in the presence of *L. fallax* silk, images of drying of sausage pieces and fresh raspberries wrapped into a treated spider web, images of the control sample for fibroblast cell adhesion tests, table of a compilation of fatty acids queried and detected by LC high-resolution time-of-flight MS (PDF)
