# Supplementary material for: Modulating Surface Properties of the Linothele fallax Spider Web by Solvent Treatment
Source: Biomacromolecules. 2021 Oct 13;22(12):4945–55. doi: 10.1021/acs.biomac.1c00787 (PMC8672351; doi:10.1021/acs.biomac.1c00787)
Supplement: Supplementary file 1 — bm1c00787_si_001.pdf [file bm1c00787_si_001.pdf]

Supporting information for

Modulating surface properties of the Linothele fallax spider web by solvent treatment

Aleksandra Kiseleva,[a] Gustav Nestor,[b] Johnny R Östman,[b] Anastasiia Kriuchkova[a], Artemii Savin[a], Pavel Krivoshapkin,[a] Elena Krivoshapkina,[a] Gulaim A. Seisenbaeva,[b] Vadim G. Kessler[b]***

[a.] Institute of Solution Chemistry of Advanced Materials and Technologies, ITMO University, St. Petersburg, Russia

[b.] Department of Molecular Sciences, Biocenter, SLU, Box 7015, 75007 Uppsala, Sweden

KEYWORDS: spider silk, thread structure, protein knots, adsorbed molecules, anti-bacterial effect

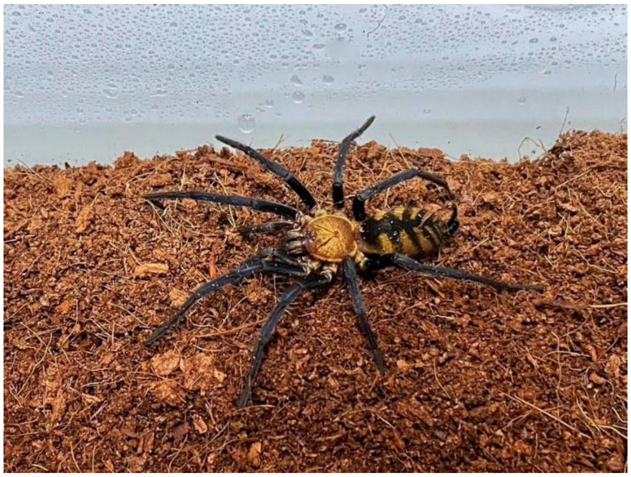

Figure S1. *Linothele fallax*, (Mello-Leitão) (LF) spider image.

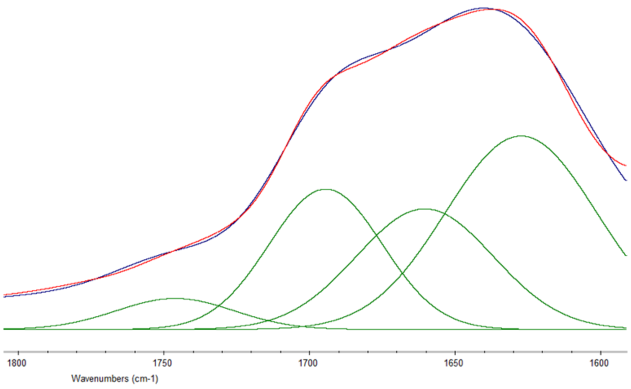

Figure S2. Process of native spider silk deconvolution in Omnic 9 software.

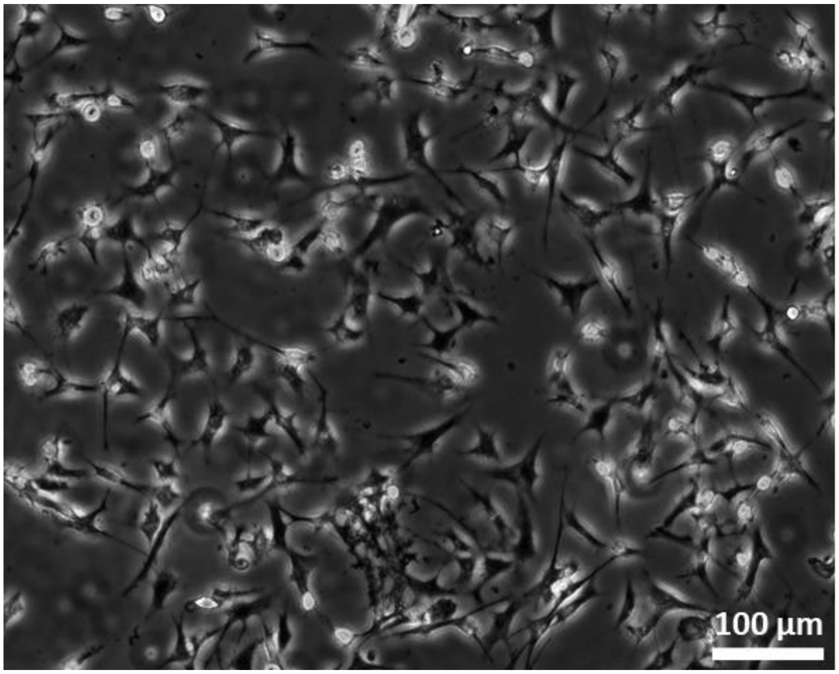

Figure S3. Image of the control sample for fibroblast cell adhesion tests.

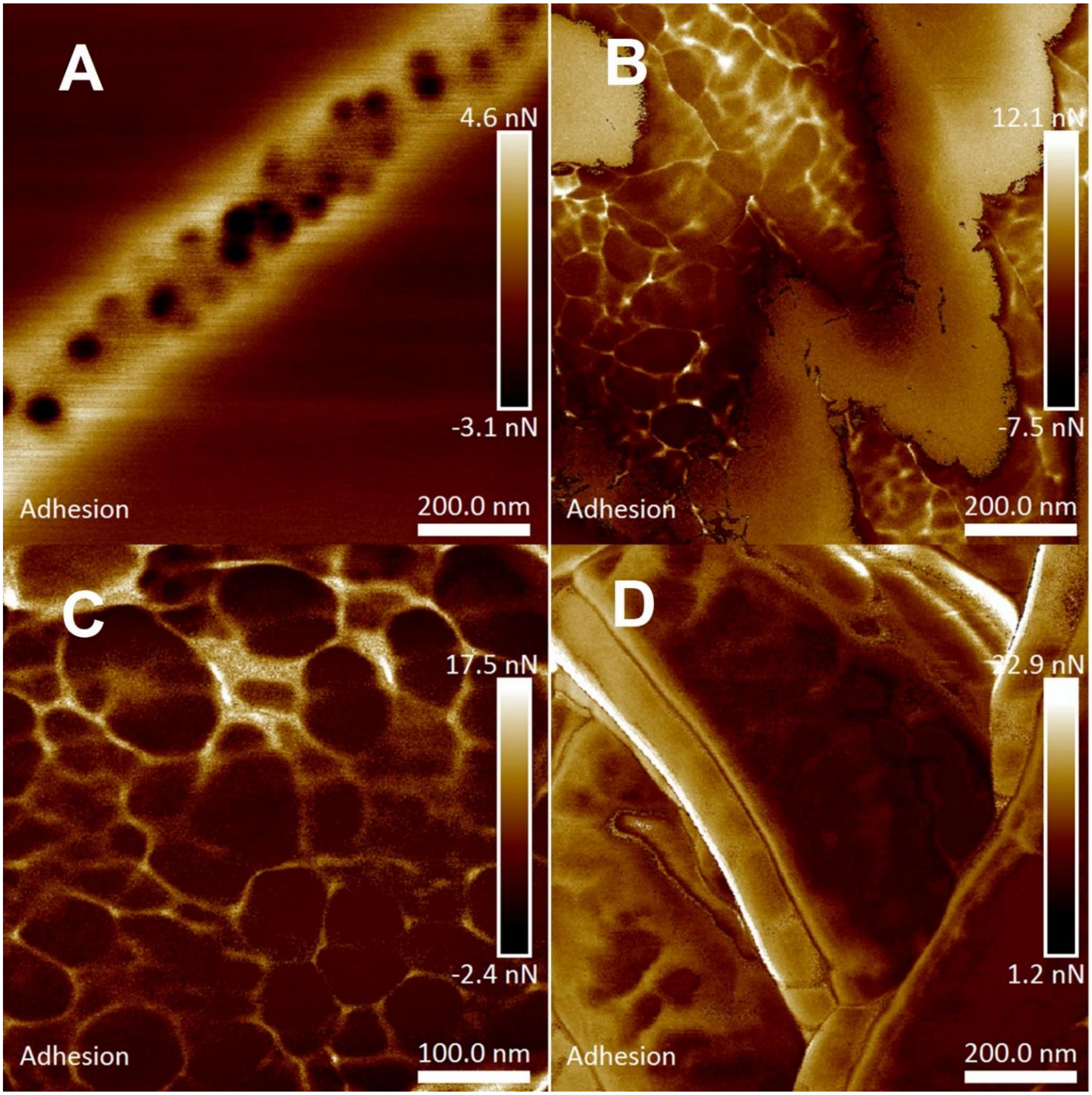

Figure S4. Adhesion force mapping of the sample: natural spider silk (A), water treated (B), ethanol treated (C) and DMSO treated (D) samples.

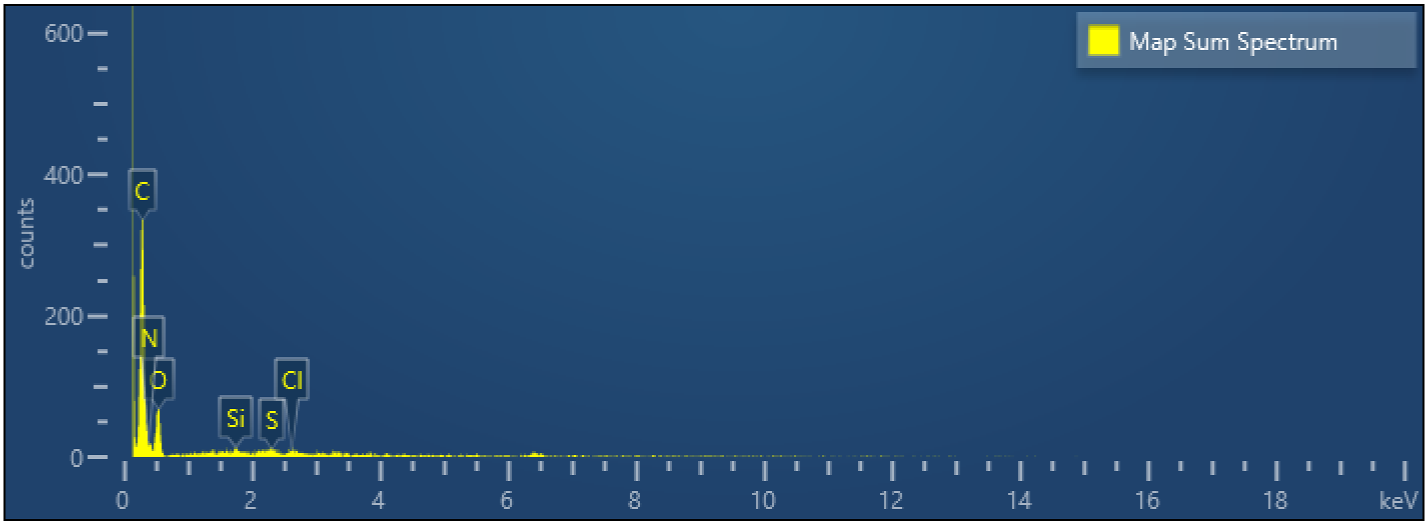

Map Sum Spectrum				
**Element**	**Line Type**	**Weight %**	**Weight % Sigma**	**Atomic %**
C	K series	51.48	1.64	56.71
O	K series	14.63	1.05	12.10
N	K series	32.61	1.94	30.81
Si	K series	0.14	0.07	0.07
S	K series	0.13	0.08	0.05
Cl	K series	0.18	0.08	0.07
Fe	K series	0.82	0.29	0.19
Total		100.00		100.00

Figure S5a. Average EDS spectrum and sum of relative contents of observed elements in untreated natural spider silk.

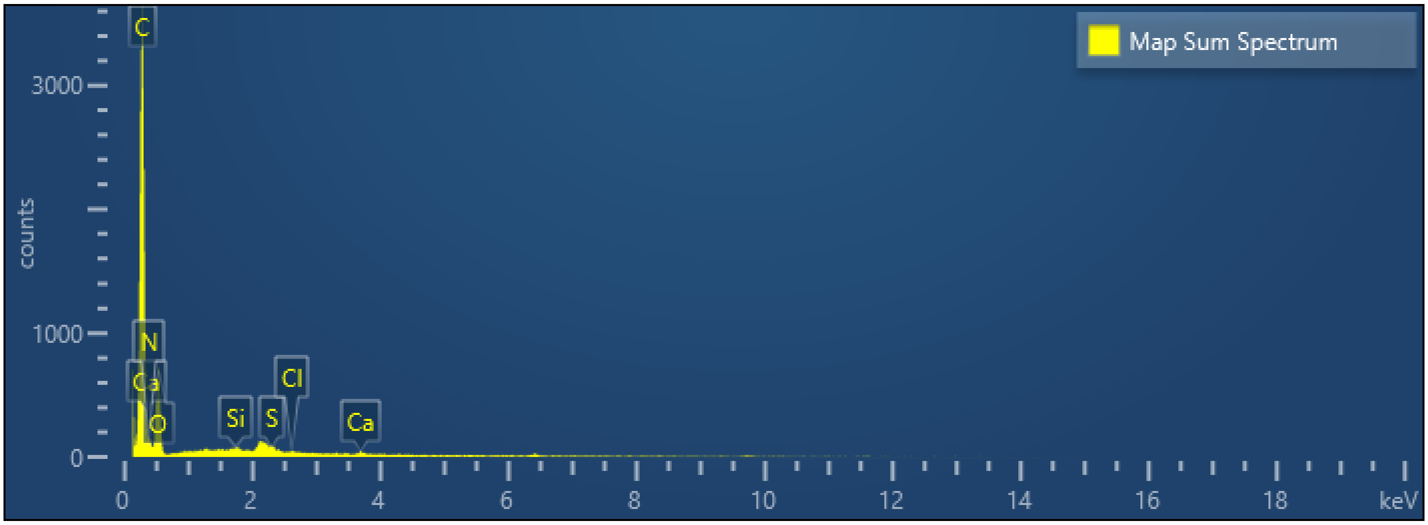

Map Sum Spectrum				
**Element**	**Line Type**	**Weight %**	**Weight % Sigma**	**Atomic %**
C	K series	53.57	1.35	59.52
O	K series	25.03	0.79	20.88
Ca	K series	0.26	0.05	0.08
Fe	K series	0.71	0.13	0.17
N	K series	20.20	1.86	19.25
S	K series	0.03	0.05	0.01
Si	K series	0.11	0.04	0.05
Cl	K series	0.09	0.04	0.04
Total		100.00		100.00

Figure 5b. Average EDS spectrum and sum of relative contents of observed elements in natural spider silk treated with EtOH.

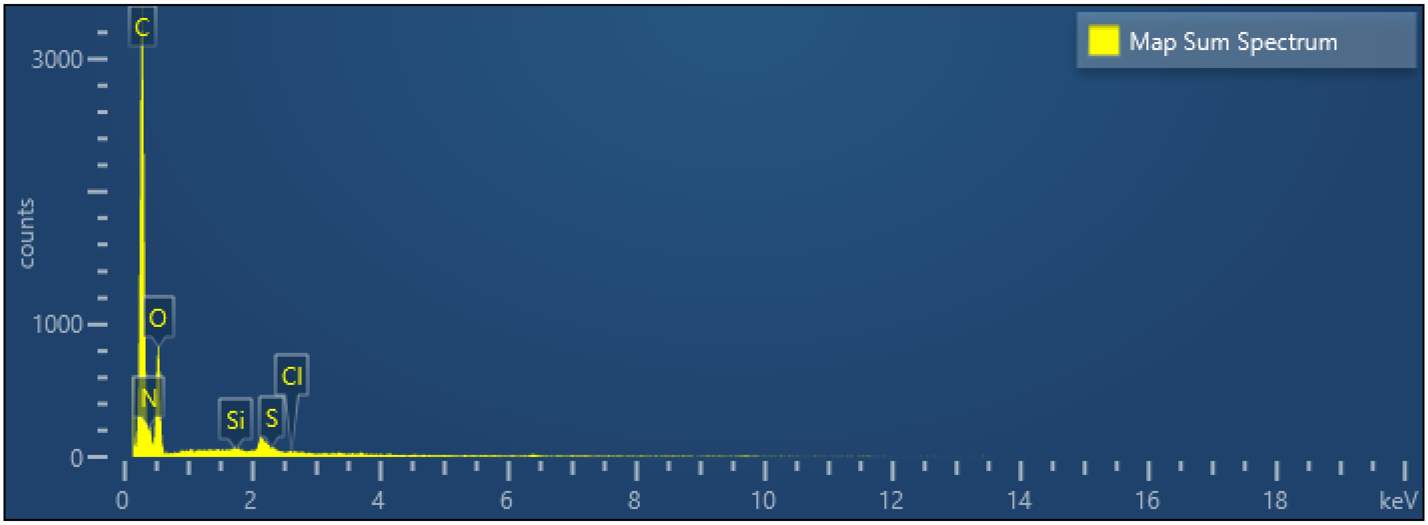

Map Sum Spectrum				
**Element**	**Line Type**	**Weight %**	**Weight % Sigma**	**Atomic %**
C	K series	51.78	1.24	57.86
O	K series	30.24	0.85	25.36
Fe	K series	0.53	0.13	0.13
Si	K series	0.06	0.04	0.03
N	K series	17.32	1.80	16.60
S	K series	0.00	0.05	0.00
Cl	K series	0.07	0.04	0.03
Total		100.00		100.00

Figure 5c. Average EDS spectrum and sum of relative contents of observed elements in natural spider silk treated with water.

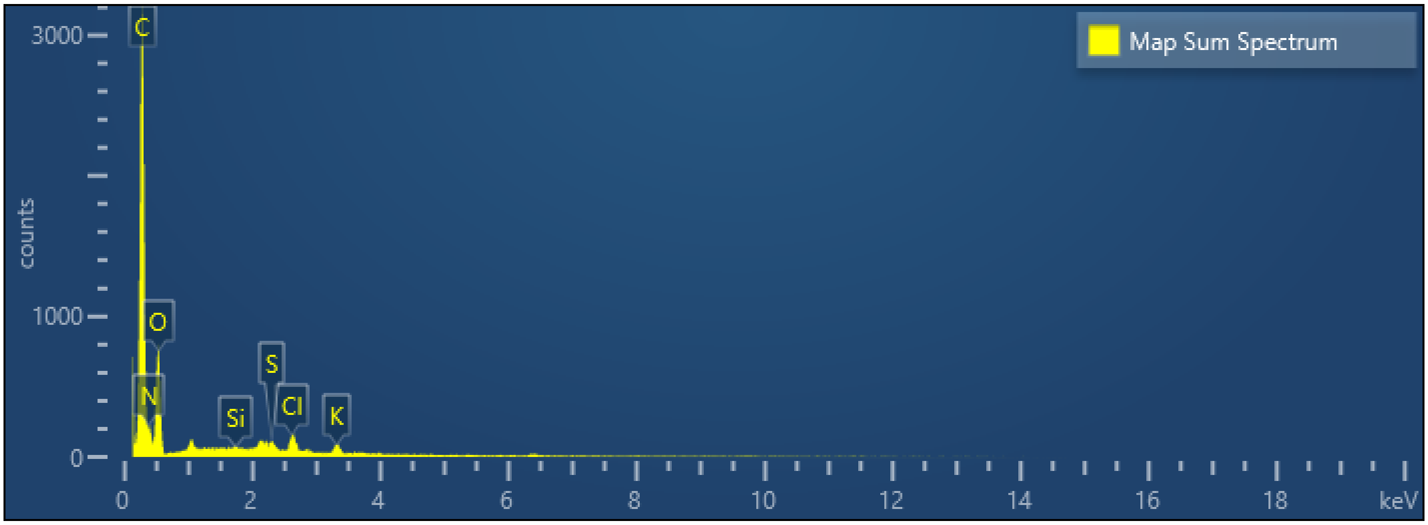

Map Sum Spectrum				
Element	Line Type	Weight %	Weight % Sigma	Atomic %
C	K series	53.31	1.14	59.62
O	K series	24.32	0.67	20.42
Cl	K series	0.82	0.06	0.31
K	K series	0.59	0.05	0.20
Na	K series	0.48	0.06	0.28
S	K series	0.18	0.05	0.08
Fe	K series	0.50	0.11	0.12
N	K series	19.76	1.50	18.95
Si	K series	0.04	0.03	0.02
Total		100.00		100.00

Figure 5d. Average EDS spectrum and sum of relative contents of observed elements in natural spider silk treated with DMSO.

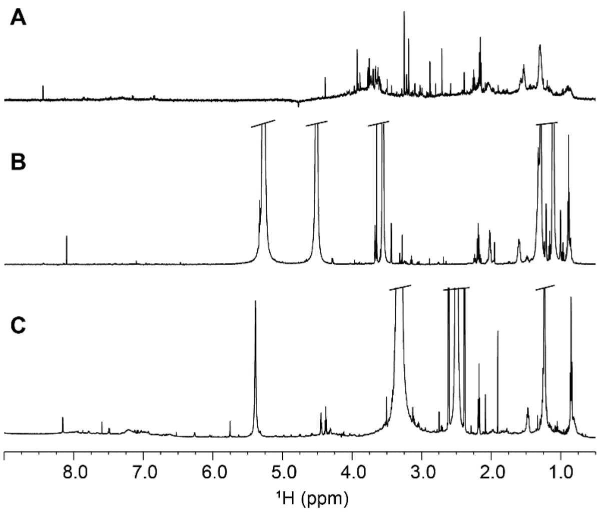

Figure S6. ^{1}H NMR spectra from A) water, B) ethanol, and C) DMSO extracts of LF spider silk.

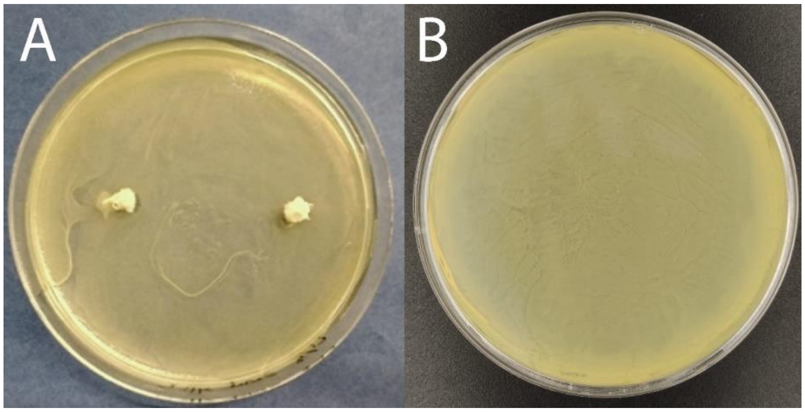

Figure S7. Image of *S. aureus* Gram-positive bacteria growth A) in the presence of LF silk, B) control image of *S. aureus* Gram-positive bacteria growth without spider silk.

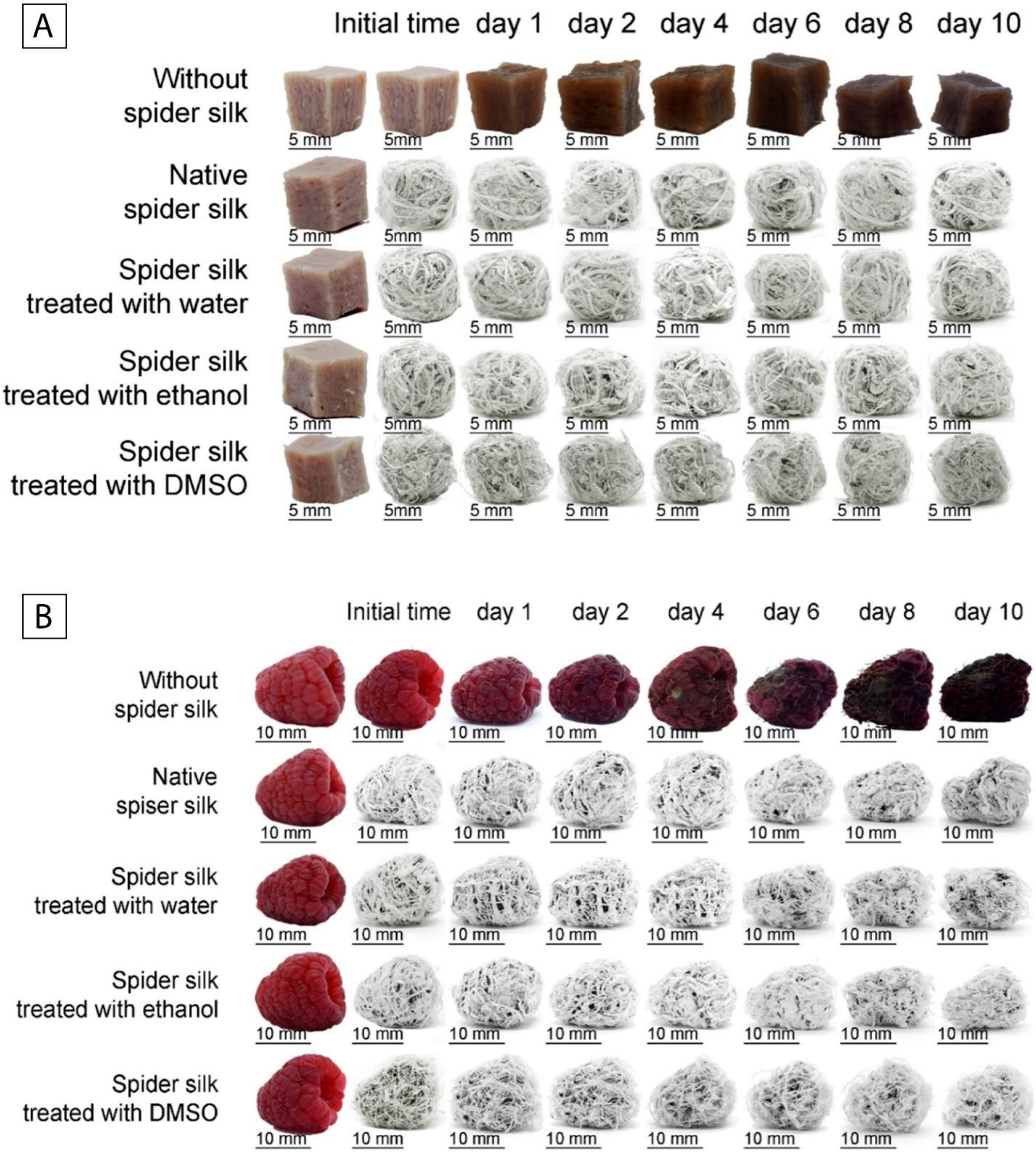

Figure S8. Images of drying of A) sausage piece and B) fresh raspberries without spider silk, wrapped into native spider silk and spider web treated with different solvents**.**

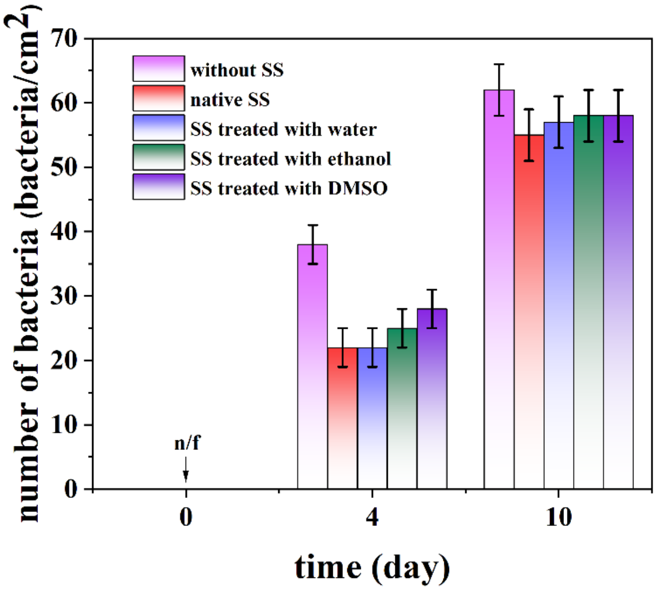

Figure S9. The number of bacteria in sausage pieces without spider silk, wrapped into native spider silk and spider web treated with different solvents. No bacteria were found in the samples at the start of the analysis. n/f – not found.

Table S1. Compilation of fatty acids queried and detected by liquid chromatography high-resolution time-of-flight mass spectrometry.

Carbons	Double bonds	Carboxylic acids	Detected *m/z*	Accurate *m/z*	Error (ppm)	RT (min)	EtOH	H_2O	DMSO
3	0	1	-	73.0295	-	-	-	-	-
4	0	1	87.0450	87.0452	2.3	4.9	X	X	X
5	0	1	101.0604	101.0608	4.0	4.9	X	X	X
6	0	1	115.0761	115.0765	3.5	4.9	X	X	X
7	0	1	129.0916	129.0921	3.9	4.9	X	X	X
8	0	1	143.1070	143.1078	5.6	4.9	X	X	X
9	0	1	157.1226	157.1234	5.1	4.9	X	X	X
10	0	1	171.1382	171.1391	5.3	4.9	X	X	X
11	0	1	185.1537	185.1547	5.4	4.9	X	X	X
12	0	1	199.1695	199.1704	4.5	4.9	X	X	X
13	0	1	213.1851	213.1860	4.2	4.9	X	X	X
14	0	1	227.2008	227.2017	4.0	4.9	X	X	X
15	0	1	241.2164	241.2173	3.7	4.9	X	X	X
16	0	1	255.2325	255.2330	2.0	4.9	X	X	X
17	0	1	269.2476	269.2486	3.7	4.9	X	X	X
18	0	1	283.2636	283.2643	2.5	4.9	X	X	X
19	0	1	297.2788	297.2799	3.7	4.9	X	X	X
20	0	1	311.2943	311.2956	4.2	4.9	X	X	X
21	0	1	325.3099	325.3113	4.3	4.9	X	X	X
22	0	1	339.3258	339.3270	3.5	4.9	X	X	X
23	0	1	353.3414	353.3427	3.7	4.9	X	X	X
24	0	1	367.3567	367.3584	4.6	4.9	X	X	X
25	0	1	381.3722	381.3741	5.0	4.9	X	X	X
26	0	1	395.3889	395.3898	2.3	4.9	X	X	X
3	1	1	-	71.0139	-	-	-	-	-
4	1	1	85.0295	85.0295	0.0	4.9	X	X	X
5	1	1	99.0448	99.0452	4.0	4.9	X	X	X
6	1	1	113.0603	113.0608	4.4	4.9	X	X	X
7	1	1	127.0756	127.0765	7.1	4.9	X	X	X
8	1	1	141.0911	141.0921	7.1	4.9	X	X	X
9	1	1	155.1069	155.1078	5.8	4.9	X	X	X
10	1	1	169.1229	169.1234	3.0	4.9	X	X	X
11	1	1	183.1382	183.1391	4.9	4.9	X	X	X
12	1	1	197.1543	197.1547	2.0	4.9	X	X	X
13	1	1	211.1698	211.1704	2.8	4.9	X	X	X
14	1	1	225.1851	225.1860	4.0	4.9	X	X	X
15	1	1	239.2002	239.2017	6.3	4.9	X	X	X
16	1	1	253.2163	253.2173	3.9	4.9	X	X	X
17	1	1	267.2318	267.2330	4.5	4.9	X	X	X
18	1	1	281.2477	281.2486	3.2	4.9	X	X	X

Carbons	Double bonds	Carboxylic acids	Detected *m/z*	Accurate *m/z*	Error (ppm)	RT (min)	EtOH	H_2O	DMSO
19	1	1	295.2631	295.2643	4.1	4.9	X	X	X
20	1	1	309.2793	309.2799	1.9	4.9	X	X	X
21	1	1	323.2941	323.2955	4.3	4.9	X	X	X
22	1	1	337.3106	337.3111	1.5	4.9	X	X	X
23	1	1	-	351.3267	-	-	-	-	-
24	1	1	-	365.3423	-	-	-	-	-
25	1	1	-	379.3579	-	-	-	-	-
26	1	1	-	393.3735	-	-	-	-	-
3	2	1	-	68.9982	-	-	-	-	-
4	2	1	-	83.0139	-	-	-	-	-
5	2	1	97.0291	97.0295	4.1	4.9	X	X	X
6	2	1	111.0446	111.0452	5.4	4.9	X	X	X
7	2	1	125.0600	125.0608	6.4	4.9	X	X	X
8	2	1	139.0756	139.0765	6.5	4.9	X	X	X
9	2	1	153.0912	153.0921	5.9	4.9	X	X	X
10	2	1	167.1069	167.1078	5.4	4.9	X	X	X
11	2	1	181.1231	181.1234	1.7	4.9	X	X	X
12	2	1	195.1384	195.1391	3.6	4.9	X	X	X
13	2	1	209.1543	209.1547	1.9	4.9	X	X	X
14	2	1	223.1689	223.1704	6.7	4.9	X	X	X
15	2	1	237.1847	237.1860	5.5	4.9	X	X	X
16	2	1	251.2001	251.2017	6.4	4.9	X	X	X
17	2	1	265.2175	265.2173	0.8	4.9	X	X	X
18	2	1	279.2319	279.2330	3.9	4.9	X	X	X
19	2	1	293.2478	293.2486	2.7	4.9	X	X	X
20	2	1	307.2633	307.2643	3.3	4.9	X	X	X
21	2	1	321.2820	321.2800	6.2	4.9	X	X	X
22	2	1	335.2935	335.2957	6.6	4.9	X	X	X
23	2	1	-	349.3114	-	-	-	-	-
24	2	1	-	363.3271	-	-	-	-	-
3	0	2	-	103.0037	-	-	-	-	-
4	0	2	-	117.0193	-	-	-	-	-
5	0	2	131.0346	131.0350	3.1	4.9	X	X	X
6	0	2	145.0499	145.0506	4.8	4.9	X	X	X
7	0	2	159.0654	159.0663	5.7	4.9	X	X	X
8	0	2	173.0813	173.0819	3.5	4.9	X	X	X
9	0	2	187.0966	187.0976	5.3	4.9	X	X	X
10	0	2	201.1125	201.1132	3.5	4.9	X	X	X
11	0	2	215.1283	215.1289	2.8	4.9	X	X	X
12	0	2	229.1435	229.1445	4.4	4.9	X	X	X
13	0	2	243.1590	243.1602	4.9	4.9	X	X	X
14	0	2	257.1741	257.1758	6.6	4.9	X	X	X
15	0	2	271.1904	271.1915	4.1	4.9	X	X	X

Carbons	Double bonds	Carboxylic acids	Detected *m/z*	Accurate *m/z*	Error (ppm)	RT (min)	EtOH	H_2O	DMSO
16	0	2	285.2059	285.2071	4.2	4.9	X	X	X
17	0	2	299.2225	299.2228	1.0	4.9	X	X	X
18	0	2	313.2371	313.2384	4.2	4.9	X	X	X
19	0	2	327.2531	327.2541	3.1	4.9	X	X	X
20	0	2	341.2687	341.2697	2.9	4.9	X	X	X
21	0	2	355.2845	355.2853	2.3	4.9	X	X	X
22	0	2	369.2999	369.3009	2.7	4.9	X	X	X
23	0	2	383.3151	383.3165	3.7	4.9	X	X	X
24	0	2	397.3318	397.3321	0.8	4.9	X	X	X
25	0	2	411.3468	411.3477	2.2	4.9	X	X	X
26	0	2	425.3624	425.3633	2.1	4.9	X	X	X
27	0	2	439.3781	439.3789	1.8	4.9	X	X	X
28	0	2	453.3938	453.3945	1.5	4.9	X	X	X
29	0	2	467.4103	467.4101	0.4	4.9	X	X	X
30	0	2	481.4253	481.4257	0.8	4.9	X	X	X
31	0	2	495.4401	495.4413	2.4	4.9	X	X	X
32	0	2	-	509.4569	-	-	-	-	-
33	0	2	-	523.4725	-	-	-	-	-
34	0	2	-	537.4881	-	-	-	-	-
4	1	2	-	115.0037	-	-	-	-	-
5	1	2	-	129.0193	-	-	-	-	-
6	1	2	-	143.0350	-	-	-	-	-
7	1	2	157.0499	157.0506	4.5	4.9	X	X	X
8	1	2	171.0656	171.0663	4.1	4.9	X	X	X
9	1	2	185.0811	185.0819	4.3	4.9	X	X	X
10	1	2	199.0964	199.0976	6.0	4.9	X	X	X
11	1	2	213.1126	213.1132	2.8	4.9	X	X	X
12	1	2	227.1275	227.1289	6.2	4.9	X	X	X
13	1	2	241.1435	241.1445	4.1	4.9	X	X	X
14	1	2	255.1591	255.1602	4.3	4.9	X	X	X
15	1	2	269.1747	269.1758	4.1	4.9	X	X	X
16	1	2	283.1905	283.1915	3.5	4.9	X	X	X
17	1	2	297.2066	297.2071	1.7	4.9	X	X	X
18	1	2	311.2208	311.2228	6.4	4.9	X	X	X
19	1	2	325.2376	325.2384	2.5	4.9	X	X	X
20	1	2	339.2521	339.2541	5.9	4.9	X	X	X
21	1	2	353.2685	353.2698	3.7	4.9	X	X	X
22	1	2	-	367.2855	-	-	-	-	-
23	1	2	381.3003	381.3012	2.4	4.9	X	X	X
24	1	2	395.3160	395.3169	2.3	4.9	X	X	X
